# Chirurgical Cases

**Published:** 1782

**Authors:** 


### LONDON MEDICAL JOURNAL,

FOR

JAN. FEB. AND MARCH, 1782.

### SECTION I.

### BOOKS.

I. *Chirurgifcke Händelfer, &c.* i. e. *Chirurgical Cafes. By* Olaus Acrel, M. D. *General Director of all the Hofpitals in Sweden; Profeffor of Surgery, and principal Surgeon to the Royal Infirmary at Stockholm. The fecond Edition.* 8vo, Stockholm, 1778. 2 Vols. with Copperplates.

THE author of the work before us, has long been defervedly efteemed as a ufeful and ingenious writer. The firft edition of it was printed in 1759, in one volume 8vo. In the prefent edition it is confiderably enlarged, and its value increaſed by many additional obſervations. The whole may be conſidered as the reſult of long and extenſive experience; the author having officiated as Surgeon to the Infirmary at Stockholm, from the time of its inſtitution in 1752. As a reward for his ſervices, the King of Sweden has lately decorated him with the order of Waſa.

The caſes deſcribed in theſe volumes are arranged under different heads. The author begins with ſuch as relate to the head and throat, and proceeds from thoſe to the diſeaſes of the trunk and extremities. From each of theſe diviſions we ſhall ſelect ſuch obſervations as appear to be the moſt intereſting.

*Wounds of the Head.*—The author relates the caſe of a ſoldier who received a violent contuſion on his head, which deprived him of his ſenſes. At the end of eight days, however, he was ſufficiently recovered to be able to walk ſeveral miles, when he ſuddenly fell down ſenſeleſs. In this ſtate he was brought to the hoſpital. No mention was made of the previous blow on the head, and as there was no wound of the integuments, recourſe was had to V.S. bliſters, and internal remedies. After he had been a week in the the hoſpital without any amendment, his head was obſerved to be enlarged, and upon examination a fluctuation was felt under the integuments. An inciſion was now made through the ſkin in the direction of the ſagittal ſuture, and by this means a conſiderable quantity of a yellowiſh watery fluid was diſcharged. Upon introducing a finger into the wound, the ſuture was found to be conſiderably dilated. The patient after this began to recover his ſpeech, and gave an account of the blow he had received. At the end of another week a fluctuation was felt at the occiput. A freſh inciſion was made in this part, and a great quantity of the ſame kind of fluid diſcharged as before. The lambdoidal ſuture was likewiſe found to be dilated, and five of the *oſſa triquetra* were ſo looſe, that they were taken out without the leaſt reſiſtance. After a few days a ſimilar diſcharge took place from the right coronal ſuture, which was alſo much dilated. In about five weeks, the diſcharge from the inciſions had entirely ceaſed, and the patient began to recover his ſtrength ſo as to be able to walk. But at this period of the cure, as he was riſing in the night to go to the privy, he accidentally fell with his head againſt the wall, and died inftantly. His head was examined after death, but the brain afforded no marks of fuppuration, or of extravafated blood, and all the futures, except the coronal, were filled with granulations.

*A cancerous fungus of the brain.*—About a year and a half before the appearance of this tumour, the patient had received a violent blow on the head, and from that period had been hardly ever free from pain. The fwelling at length acquired the fize of a middling apple. As it evidently rofe and fubfided in a manner correfponding with the motion of the cerebrum, it was on this account fuppofed to be connected with it. It gradually became fofter and fofter, and at length was opened. A confiderable quantity of a thin bloody fluid was difcharged by this means, but there was no appearance of pus. Soon after the operation the patient fell afleep, and remained fenfelefs and motionlefs till his death, which happened three days afterwards.

This cafe is followed by an account of two inftances of a peculiar tumour on the head. In both patients this fwelling was brought on by their carrying a great weight on the head. It extended over the whole cranium to the *os jugale.*

*gale.* It was neither red nor painful, but very elaſtic, ſo as not to retain the impreſſion of the finger. In each of theſe caſes a cure was eaſily obtained by evacuations and topical applications, as the cranium did not ſeem to be affected.

*A double hare-lip.*—In this caſe the author truſting to the great dilatability of the *labia*, cut out the portion of hard fleſh that ſeparated the two hare-lips, and which was 4-10ths of an inch in breadth. The event was perfectly ſucceſsful.

*Cancer of the lower lip.*—In caſes of this ſort, if any part of the cheek is indurated or corroded, or the neighbouring glands are enlarged, he conſiders the diſeaſe as incurable. Several caſes are related, in which the Belladona, Phytolacca, and other medicines were of no uſe. In ſeveral of theſe caſes, the operation proved fruitleſs: for although the wound healed kindly, either the ſubmaxillary glands, or the glands of the neck ſwelled, and proved fatal to the patient. In ſome inſtances, however, he has ſeen the operation prove ſucceſsful.

*Of the Trichiaſis.*—The author obſerves that this is generally the effect of chronic ophthalmia. In moſt of the caſes that have occurred to him, the patient was deprived of ſight; but in all of them them a cure was effected by cutting out a portion of the outer membrane of the eye-lids, which was always præternaturally elongated and relaxed.

*A large tumour extracted from the orbit of the eye.*—This tumour was of the ſize of an hen's egg, and aroſe from the bottom of the orbit, ſo that the bulb of the eye was nearly preſſed out of that cavity. After making an inciſion thro' the integuments above the orbit, the tumor was extracted, and the optic nerve was clearly ſeen after the operation. By degrees, however, the eye reſumed its natural form, and the patient recovered his ſight.

*A cancerous eye ſucceſsfully extirpated.*—The ſubject of this caſe was a boy, and the diſeaſed eye had acquired an enormous bulk. Our author firſt made an inciſion, of an inch in length, through the canthus externus, and after diſſecting the bulb from the eye-lids, paſſed a thread through it, by which means he was enabled to elevate and extract it with eaſe. The orbit by degrees was filled with a fleſhy ſubſtance, and five weeks after the operation, the patient was perfectly recovered. The extirpated eye afforded no marks of organiſation.

Of

*Of the cataract.*—In cafes of this fort, Dr. Acrel prefers depreffion to extraction, and the method recommended by St. Ives to that practifed by Ferrein. He has examined, after death, the eyes of feveral perfons who in their lifetime had undergone the operation of depreffion, but in none of them could he difcover the leaft veftige of cataract in the chamber of the eye. He therefore is of opinion that the depreffed lens is gradually diffolved, and of courfe difappears. He obferves that of thirty cafes of cataract, there were only three in which the anterior capfula of the lens was tranfparent; in all the others it was opaque. He has hardly ever feen any bad confequences from an effufion of blood into the eye during the operation, as it commonly foon difappeared. Repeated obfervations have convinced him, that in cafes where the cataract has been preceded by long continued pains or defluxions, or rheumatic headach, and the face is puftulous, or of a copper-colour, the operation is generally followed by bad fymptoms, and the patient feldom recovers his fight.

*Polypus of the nofe.*—Dr. Acrel obferves, that the hard flefhy polypus has very often a bony bafis, baſis, and is not radically cured 'till an exfoliation has taken place. In order to promote this, he applies butter of antimony, through a tube, to the part, and by this means has always deſtroyed the root of the polypus, and prevented it from growing again.

*Extirpation of the parotids.*—Two caſes are related in which this operation was performed by firſt making a crucial inciſion through the integuments, and then paſſing a needle and thread through the tumour, by means of which it was drawn upwards, and diſſected out with greater eaſe. In one of the patients, the extirpation was effected without difficulty; in the other, it was attended with conſiderable trouble and danger, owing to the wounding of the external carotid. In leſs than two minutes the patient loſt upwards of two pounds of blood. The artery was too deeply ſeated to allow of a ligature being paſſed round it, and our author was afraid to apply ſtyptics on account of the numerous branches of nerves that occur in that part. There was no time, however to be loſt, and recourſe was had to agaric, pieces of whi h were applied ſo as to compreſs the mouth of the artery, and this had the deſired effect.

*A carious fiftula in the meatus auditorius.*—The difeafe here fpoken of came on after an acute rheumatifm, fucceeded by vertigo, watchfulnefs, violent head-ach, and the difcharge of a yellowifh, watery fluid of a four fmell, from the ear. The meatus auditorius was found filled with a foft fpongy flefh, and upon introducing a probe into it, our author felt a piece of loofe rough bone. This induced him to dilate the meatus, and extract the difeafed bone, which was accordingly done, and the patient afterwards recovered.

*Worms in the meatus auditorius*,—The fubject of this cafe was a female who had for fome time laboured under a difficulty of hearing. On a fudden without any apparent caufe, fhe was feized with convulfions, and foon afterwards complained of acute pain in her ears, upon which the convulfions returned with increafed violence. A piece of lint moiftened with oil and laudanum, was now put into the ear, and upon taking it out, feveral very fmall round worms were found upon the lint, whereupon all the fymptoms ceafed. The author informs us that he has fince found an excellent remedy againft worms of this kind in a decoction of

*Ledum*

*Ledum paluſtre* injected into the ear, having experienced its efficacy in ſeveral caſes.

*A piece of a tobacco-pipe diſcharged from an abſceſs.*—The patient whoſe caſe is here related, fell aſleep with a pipe in his mouth, and broke off a portion of it, which he ſwallowed. From that time he was ill, and ſoon began to have ſymptoms of hectic fever. Several months after an abſceſs formed in his back under the lower angle of the ſcapula, and on opening it, it was found to contain the piece of the tobacco-pipe, which was eaſily extracted, and the patient cured.

*Caſe of a gun-ſhot wound in the throat.*—In this patient the ball paſſed between the trachea and the left internal carotid, without ſeeming to have injured either of thoſe parts. Towards the end of the cure a true aneuriſm of the carotid, of the ſize of a walnut, made its appearance, but was perfectly removed by compreſſion in leſs than ſix months.

*Encyſted tumours.*—In ſeveral caſes of this ſort, the tumours were opened by inciſion, and the ſac deſtroyed by ſuppuration. When the tumours have been large, Dr. Acrel has obſerved, that ſmall ſinuſes and ſacs have ſometimes remained mained between the muſcles, and afterwards filled again. When this happened a ſecond opening generally proved effectual. If the diſcharge from the wound is ichorous, he recommends the application of a ſolution of ſulphur, as the beſt means of procuring a laudable pus. He has ſeen hard encyſted ſwellings brought to ſuppuration by the external uſe of Pott's *Arthritic Spirit*, which is prepared by diſtilling two ounces of common ſalt with one ounce of oil of vitriol and two of oil of turpentine. The diſtilled ſpirit is to be carefully ſeparated from the water, and preſerved for uſe.

*Caſes of vomica.*—Our author has ſeen repeated inſtances of abſceſſes of this ſort after pleuriſy. The patients had commonly purulent expectoration, and external fiſtulous ulcers. He always thought it adviſeable to begin by dilating the external opening. In the greater number of caſes, he obſerved that the pus was not in the cavity of the thorax, but in a peculiar ſac between the pleura and intercoſtal muſcles; ſometimes, however, it was evidently in the cavity; and the caſes in which this happened were generally found to be the moſt fatal.

*Abſceſs of the liver.*—The ſuppuration here ſpoken of took place after a violent hepatitis. The firſt appearance of it was a large ſwelling in the right hypochondriac region, extending to the breaſt: the patient had a weak, quick pulſe, his countenance was of a pale yellow, and he was much emaciated. Poultices were applied to the tumour, and in ſix days the fluctuation was ſufficiently evident to allow of its being opened. A large quantity of yellowiſh green pus was diſcharged, and a cure was obtained in about ſeven weeks.

*Of hernias.*—Under this head we meet with ſeveral intereſting obſervations.—In a caſe of inveterate incarcerated hernia, our author dilated the abdominal ring before he opened the hernial ſac, and the inteſtine immediately went up of itſelf. He did this, he tells us, to prevent the air from coming into immediate contact with the contents of the ſac. After the reduction of the hernia, the ſac was opened, and the patient recovered, and remained well for nine months; at the end of which time his irregular mode of living brought on a colic, of which he died. Upon diſſection thoſe parts of the omentum and inteſtine which had formerly deſcended defcended through the ring, were found con-nected into one lump; the omentum was preternaturally hard, and the membranes of the inteftine much thickened. The diameter of the fac at the ring was fo very fmall, that a goofe quill could with difficulty be paffed into it.

In feveral cafes of hernia that were not incarcerated, the author has performed the operation with a view to prevent incarceration. Some of the patients were radically cured; in others, the hernia appeared again when they left off wearing a bandage; and in one cafe the operation proved fatal. In favour of this mode of practice, he obferves that if the patient is of a good habit of body, and the inteftines are fpeedily reduced, the operation is not a dangerous one; and that in old hernias it often happens that the patient, even with the affiftance of a trufs, is unable to prevent the hernia from coming down, and of courfe is every moment in danger of its being incarcerated; whereas by means of the operation a contraction of the ring is produced, fo that the hernia can always be kept up with the affiftance of a bandage.

A caſe of incarcerated hernia is related in which our author performed the operation: a large portion of the omentum was found in the ſac, to the baſis of which it adhered. After dilating the abdominal ring, the omentum was eaſily reduced, and the patient recovered. In three months after the operation, the omentum could be felt above the ring, but after that period it entirely diſappeared.

*Separation of the oſſa pubis in parturition.*—This circumſtance was not obſerved till five weeks after delivery, when upon opening an abſceſs that had formed in the pubis, it was diſcovered that the bones were ſeparated, and eroded by the pus. After an exfoliation of the carious part, the bones united again, and the patient recovered.

*Of fiſtulas of the urethra.*—Theſe complaints, the author remarks, are generally, though not always the effect of a venereal cauſe. In proof of this he relates the caſe of a ſoldier, who, after an acute fever, was afflicted with an inflammation of the teſticles, ſcrotum, and perinæum. A ſuppuration took place in the latter, and the urine made its way through the wound. In this ſituation he remained for ten years, when he he was received into the hoſpital. At that time there were three fiſtulas *in perinæo*; one at the lower part of the ſcrotum, and two others near the anus. Almoſt the whole of his urine paſſed through theſe channels. Recourſe was had to bougies, and mercurial ointment was applied to the perinæum. In a ſhort time the bougies procured a free diſcharge of urine from the natural paſſage, but the perinæum remained hard as before. A probe was introduced into the urethra, and an inciſion made into it. Several other inciſions were at the ſame time made into other parts of the perinæum. A good ſuppuration enſued; the hardneſs diſappeared; and in about five weeks after this operation the whole was cloſed by a ſoft cicatrix. The patient was now diſcharged from the hoſpital, with a caution to continue the uſe of the bougies. This advice however he neglected to follow, and at the end of two years, returned to the hoſpital with a freſh induration of the perinæum, and voiding his urine with difficulty, and only by drops. The hardneſs ſeeming to be occaſioned by a fiſtula near the neck of the bladder, an inciſion was made into the urethra, and the indurated part diſſected out: after which bougies were ufed, as after the former operation, and the patient foon recovered.—The author afcribes the origin of this cafe to a metaftafis after fever; but we are inclined to think that he was deceived by the patient, who conftantly denied that he had any venereal taint.

*Hydrocele.*—Two inftances are mentioned in which the operation by incifion proved fatal. The author attributes his ill fuccefs to the irregularity of the patients. He advifes the operation of tapping, in cafes of this fort, to be performed in a dark room, with a lighted candle placed at the oppofite fide of the fcrotum. By this means, we are told, it is eafy to diftinguifh, and of courfe to avoid wounding the large blood-veffels of the tunica vaginalis or the tefticles.

In feveral cafes of hydrocele he has adopted Mr. Elfe's method of cure by cauftic, and has conftantly found it fuccefsful.

*Calculous complaints.*—He has given the *uva urfi* in large dofes, and to a great number of patients, but without any good effect. Of twenty-two patients whom he cut for the ftone, five died within a month after the operation; all the others recovered. In the dead body of a man who who during his life-time had been troubled with all the fymptoms of ftone, no calculus was found on diffection, but a fac was formed at the pofterior part of the bladder, refembling the *inteftinum cœcum.*

In the operation of lithotomy, in adults as well as young fubjects of both fexes, he has with great fuccefs employed Frere Cofme's *lithotome caché*, and likewife M. Le Dran's *Couteau en rondache.*

*Of Aneurifms.*—Several cafes are related, and one of them proves, that we cannot always depend on the ligature of the artery, as in the cafe alluded to, the thread was unexpectedly diffolved eight days after the operation, and the patient bled to death.

*A particular kind of Paronychia.*—Four cafes of this fort have occurred to our author. The firft of thefe patients began in the fummer of 1746 to feel an acute pain in the little finger of his left hand. The pain extended from the root of the nail to the end of the finger, and at firft lafted only a few minutes at a time, returning irregularly after intervals fometimes of feveral weeks. In about a year it became more violent, and continued for half and even for a hour. In 1748 this complaint became ftill more troublefome, and the fame fort of pain began to extend to the hand. During the years 1749 and 1750 the pain in the finger and hand increafed, returning more frequently, and continuing longer. The external ufe of theriaca and brandy fometimes alleviated it, but did not prevent its return. In the winter of 1751 the patient began to feel a fimilar pain in his arm, and the finger was extremely painful when touched. The year following, the pain in the finger, hand and arm, became much worfe, and he began at times to complain of a pungent pain in the fide under the arm affected. Externally, nothing preternatural appeared. Electricity, blifters to the finger, a vapour bath, and other remedies were tried without effect. At laft it was refolved to amputate the laft phalanx of the little finger. The operation was accordingly performed, and the pain in the hand and arm gradually went off. The amputated bone was found changed into a real fatty fubftance.

In another patient, a middle aged woman, an almoft conftant pain was felt for nine years in the laft phalanx of the right fore-finger. During the two laft years, the extremity of the finger thickthickened and became almoſt tranſparent. A cure was obtained, as in the former caſe, by amputation. The bones of the amputated phalanx were tranſparent, and of a gelatinous conſiſtence.

*A caſe in which the tendo Achillis was twice divided.*—The ſubject of this caſe was a man who had the *tendo Achillis* cut through by a broad ſword. The divided portions of the tendon were united by ſuture, and after a time the patient ſeemed to be perfectly recovered. Several years after this, as he was running haſtily up ſtairs, the tendon was again ruptured at the ſame place as before. The divided portions were now brought together by bandage. At the end of ſix weeks they ſeemed to be completely reunited, and in leſs than half a year the patient had recovered the perfect uſe of his foot. At the place where the re-union took place, a hard knot was to be felt of the ſize of a hazle-nut.

Beſides the caſes and remarks of which we have already given an abſtract, there are ſome others of which it will be ſufficient to mention the title or general heads. Of this number are 1. the caſe of a woman who voided aſcarides with her urine. 2. An inſtance of a double uterus illuſ-

trated by an engraving; 3. a case of a suppression of urine in which the bladder was ruptured by a violent effort to empty it. 4. An account of a wound of the femoral artery, in which a ligature was succesfsfully passed round the vessel, about four inches below Pouparts ligament, and the use of the limb preserved; 5. a case of a fracture of the *os humeri*, in which a spurious articulation was formed merely by neglecting to suspend the fore-arm; the latter by its weight having drawn down the lower part of the os humeri, so as to prevent it from uniting with the upper portion. 6. An account of a considerable swelling of the hand, occasioned by the sting of a wasp, and cured by the external use of cold water.

